# Absence of N-Acetylglucosamine Glycosylation on *Listeria monocytogenes* Wall Teichoic Acids Promotes Fatty Acid Tolerance by Repulsion From the Bacterial Surface

**DOI:** 10.3389/fmicb.2022.897682

**Published:** 2022-05-12

**Authors:** Rikke S. S. Thomasen, Patricia T. dos Santos, Eva M. Sternkopf Lillebæk, Marianne N. Skov, Michael Kemp, Birgitte H. Kallipolitis

**Affiliations:** ^1^Department of Biochemistry and Molecular Biology, University of Southern Denmark, Odense, Denmark; ^2^National Food Institute, Technical University of Denmark, Kgs. Lyngby, Denmark; ^3^Department of Clinical Microbiology, Odense University Hospital and Research Unit of Clinical Microbiology, University of Southern Denmark, Odense, Denmark; ^4^The Regional Department of Clinical Microbiology, Zealand University Hospital, Koege, Denmark

**Keywords:** *Listeria monocytogenes*, wall teichoic acid, antimicrobial fatty acids, anti-virulence activity, N-acetylglucosamine glycosylation

## Abstract

Free fatty acids (FFAs) have strong antimicrobial properties against pathogenic bacteria and are known as natural protective agents against bacterial infections. Growth of the foodborne pathogen *Listeria monocytogenes* is highly affected by the presence of antimicrobial FFAs, however, the response of *L. monocytogenes* toward FFAs is not fully understood. Here, we explore how *L. monocytogenes* gains tolerance toward FFAs and present a novel mechanism conferring bacterial protection against FFA toxicity. Strains tolerant against the antimicrobial FFA palmitoleic acid were isolated and whole genome sequenced, and mutations were found in genes involved in wall teichoic acid (WTA) glycosylations. We show that mutation or deletion of *lmo1079*, which is essential for N-acetylglucosamine (GlcNAc) glycosylation of WTAs, confer tolerance against several antimicrobial FFAs. The FFA tolerant strains are lacking GlcNAc on their WTAs, which result in a more hydrophilic surface. In line with this, we observed a reduced binding of FFAs to the surface of the FFA tolerant strains. Additionally, lack of GlcNAc on WTAs confers tolerance toward acid stress. Altogether, these findings support that GlcNAc modification of WTA plays an important role in the response of *L. monocytogenes* toward stress conditions encountered during growth as a saprophyte and pathogen, including FFA-rich environments. Most importantly, our data revealed that *L. monocytogenes* strains lacking GlcNAc on their WTAs are protected against FFA toxicity, because the FFAs are repulsed from the bacterial surface of GlcNAc-deficient strains.

## Introduction

Production of free fatty acids (FFAs) are known as a natural protection strategy against bacterial infections in humans, animals, and plants (Kengmo Tchoupa et al., 2021). In humans, FFAs are present in multiple areas, such as the skin and the gastrointestinal tract, where their antimicrobial properties protect the host against bacterial infections (Chatterjee et al., 2007; Kohler et al., 2009). Although FFAs are known to inhibit bacterial growth, the mechanism underlying their antimicrobial activity is not fully understood. In general, FFAs are thought to interfere with biological processes in the membrane, causing reduced nutrient uptake, cell lysis, leakage of cell metabolites, or disruption of the electron transport chain (Desbois and Smith, 2010). To cope with antimicrobial FFAs in FFA-rich environments, bacteria have evolved various strategies to avoid FFA toxicity (Kengmo Tchoupa et al., 2021). Those strategies primarily involve repulsion of FFAs from the bacterial surface, detoxification of the FFAs, or efflux of FFAs from the cytosol to the extracellular environment (Kengmo Tchoupa et al., 2021). For some pathogens, the presence of wall teichoic acids (WTAs) on the bacterial surface is essential to retain tolerance toward FFAs, e.g., those found on the skin (Kohler et al., 2009). Additionally, some bacteria can induce the expression of specific surface proteins in stressful environments, which then decrease surface hydrophobicity and cause repulsion of the FFAs (Clarke et al., 2007). By using such mechanisms, bacteria manage to protect themselves against FFAs in the environment.

*Listeria monocytogenes* is a facultative intracellular pathogen causing life-threatening foodborne diseases in susceptible individuals. During growth as a saprophyte and pathogen, *L. monocytogenes* is exposed to FFAs in various environments, including food products such as salmon and milk, and in the gastrointestinal tract of a host (Petrone et al., 1998; Chatterjee et al., 2007; Cascant et al., 2018). Upon ingestion of contaminated food, *L. monocytogenes* adapts to the gastrointestinal conditions and initiates internalization into intestinal epithelial cells through the surface protein Internalin A (InlA). When inside the phagosome, the bacterium gains access to the cytoplasm of the host cell by secretion of the pore forming virulence factor Listeriolysin O (LLO). *Listeria monocytogenes* then proliferates inside the cytosol and spreads to neighboring host cells using an actin comet tail generated by the help of Actin assembly-inducing protein (ActA; Freitag et al., 2009). The expression of virulence factors required for *L. monocytogenes*’ intracellular lifestyle is controlled by the virulence regulator PrfA (Scortti et al., 2007). We recently discovered that at low doses, specific FFAs act to repress transcription of PrfA-dependent virulence genes by inhibiting the DNA-binding activity of PrfA (Sternkopf Lillebæk et al., 2017; Dos Santos et al., 2020). At high doses, some FFAs act to prevent the growth of *L. monocytogenes*, but the response of this pathogen to the antimicrobial activity of FFAs is presently unclear.

In this study, we aimed to explore how *L. monocytogenes* responds to and copes with the antimicrobial activity of FFAs. More specifically, we studied the molecular mechanism underlying the response of *L. monocytogenes* to the antimicrobial monounsaturated FFA palmitoleic acid (PA, C16:1). PA is found in different oils from both macadamia nuts and sea buckthorn and was chosen for this study because it has a dual effect on *L. monocytogenes*: at high doses, PA acts to inhibit growth of *L. monocytogenes*, whereas at low doses, PA inhibits the activity of a PrfA mutant variant, PrfA*, that is known to be locked in a constitutively active conformation (Sternkopf Lillebæk et al., 2017; Dos Santos et al., 2020). Curiously, a mutant variant deleted for *prfA* is tolerant to PA, suggesting that PrfA also plays a role in the response to the antimicrobial activity of PA. To improve our understanding of how *L. monocytogenes* reacts to FFAs, we isolated mutant strains that are tolerant toward the antimicrobial activity of PA. Interestingly, when the PA tolerant strains were whole genome sequenced, we found a nonsense mutation in *lmo1079* in four out of six PA tolerant mutants. The *lmo1079* gene encodes for an YfhO homolog, which is essential for the N-acetylglucosamine (GlcNAc) glycosylation of the WTAs (Rismondo et al., 2018). Notably, the *L. monocytogenes* strain used in this work belongs to serotype 1/2a, which contains two substitutions on their WTAs: GlcNAc and rhamnose (Rha; Kamisango et al., 1983). Here, we present our investigations on how the lack of GlcNAc glycosylation of WTAs contributes to FFA tolerance in *L. monocytogenes*.

## Materials and Methods

### Bacterial Strains and Growth Conditions

The wild-type *Listeria monocytogenes* EGD 1/2a strain (obtained from W. Goebel, University of Wurzburg, Wurzburg, Germany) was used in the study, together with its two isogenic derivates ∆*prfA* (Böckmann et al., 1996) and prfA* (expressing a constitutive active PrfA mutant, G155S; Sternkopf Lillebæk et al., 2017). For construction of *lmo1079*-sub1, *lmo1079-*sub2, *lmo1083*-sub, and in-frame deletion mutants ∆*lmo1079* and ∆*lmo1083*, the corresponding primers (P1, P2, P3, and P4) were used (Supplementary Table S1). The fragments were amplified using a two-step PCR procedure and inserted into the temperature sensitive shuttle vector pAUL-A (Schäferkordt and Chakraborty, 1995). For *lmo1079*-sub1 complementation mutants (*lmo1079*-sub1::*lmo1079*-sub1-c and *lmo1079*-sub1-*lmo1083-*sub::*lmo1079*-sub1-c), P1 and P4 for *lmo1079*-sub1 were used for PCR amplification followed by integration into pAUL-A. The resulting pAUL-A plasmid constructs were transformed into competent *L. monocytogenes* as previously described by (Mollerup et al., 2016). Integration and disintegration of the plasmids into the bacterial genome occurred based on homolog recombination as described in (Christiansen et al., 2004). The double mutant, *lmo1079*-sub1-*lmo1083*-sub, was constructed in a two-step process, where the mutations were introduced into the bacterial genome consecutively. The resulting mutants were validated by PCR and sequencing using primers P5 and P6 flanking the mutated regions (Supplementary Table S1). p*hly-lacZ*, containing the promotor region of *hly* transcriptionally fused to *lacZ*, and p*lhrA36-lacZ*, containing the promotor region of the *lhrA* core promotor transcriptionally fused to *lacZ*, were previously constructed (Larsen et al., 2006; Nielsen et al., 2011). *Listeria monocytogenes* was routinely grown at 37°C with aeration in Brain Heart Infusion medium (BHI, Oxoid) and supplemented with 50 μg/ml kanamycin (Kan) or 5 μg/ml erythromycin (Erm) when appropriate. During cloning in pAULA, *Escherichia coli* TOP10 (Invitrogen) was used and grown at 37°C with aeration in Luria Bertani broth (LB, Sigma-Aldrich) supplemented with 150 μg/ml Erm.

### Free Fatty Acids

The following FFAs were included in this study: palmitoleic acid (PA; C16:1; Sigma-Aldrich, purity ≥98.5%), palmitic acid (PAL; C16:0; Sigma-Aldrich, purity ≥99%), lauric acid (LA; C12:0; Sigma-Aldrich, purity ≥98%), and eicosapentaenoic acid (EPA, C20:5; Sigma-Aldrich, purity ≥99%). Ninety-six percent ethanol was used as vehicle to dissolve the FFAs.

### Promotion of PA Tolerant Strains

Overnight (ON) cultures of prfA* were diluted to OD_600_ = 0.0002 in BHI and stressed with increasing concentrations of PA (2, 4, 8, 16, 32, 64, and 125 μg/ml) for 7 days. The concentration of vehicle was kept constant at 0.25% during the selection process. Glycerol stocks were made at 125 μg/ml PA, and a total of six single mutants were isolated from three biological replicates for further studies.

### Growth Experiment With FFAs

Overnight (ON) cultures were diluted to OD_600_ = 0.0002; 4 ml of the diluted cultures were transferred to culture tubes and stressed with increasing concentrations of FFAs. As controls, one culture was left untreated, and another only exposed to the vehicle. Cultures were incubated for 20 h, followed by OD_600_ measurements.

### Growth Experiments in 96-Well Plates

Overnight (ON) cultures were diluted to OD_600_ = 0.2 and 5 μl of the diluted cultures were added to their corresponding wells in the TC 96-well plate (standard, F, SARSTEDT). A final volume of 200 μl was obtained in each well by adding 195 μl BHI ± stress agents to the corresponding wells. An initial OD_600_ of 0.005 was thereby obtained. The 96-well plate was incubated in the plate reader (Synergy™ H1 multi-mode microplate reader, BioTek) for 24 h at 37°C with orbital shaking for 15 s every 30 min.

### **β**-Galactosidase Assay

ON cultures of the strains containing either p*hly-lacZ* or p*lhrA36-lacZ* were diluted to OD_600_ = 0.02. At OD_600_ = 0.3 the cultures were split and treated with either 2 μg/ml PA or 150 μg/ml PAL. As controls, a culture was left untreated, or treated with only vehicle corresponding to the final concentration of vehicle in FFA treated samples. After 20 h of growth OD_600_ was measured and 1 ml samples were harvested. The β-galactosidase assay was performed as previously described (Christiansen et al., 2004).

### Cell Harvest, RNA Extraction, and Northern Blot Analysis

Overnight (ON) cultures of prfA*, PA-1A, PA-2A and PA-3A were diluted to OD_600_ = 0.02 and incubated. At OD_600_ = 0.3 the cultures were split and treated with either vehicle or 2 μg/ml PA for 1 h. Next, cultures were snap cooled in liquid nitrogen followed by centrifugation at 8,000 rpm for 3 min at 4°C. The supernatant was removed, and pellet was snap cooled in liquid nitrogen before samples were stored at −80°C. Total RNA was extracted using Tri reagent (Molecular Research Center, Inc.), as previously reported (Nielsen et al., 2010). RNA purity and concentration were determined by agarose gel electrophoresis and DeNovix DS-11 Fx, respectively. Agarose northern blot analysis was performed as early described (Dos Santos et al., 2020). Membranes were hybridized with ^32^P-labeled single stranded probes (Supplementary Table S1). Visualization of bands was done using Typhoon FLA9000 (GE Healthcare).

### Whole Genome Sequencing

Sequencing libraries were prepared for prfA*, PA-1A, PA-1B, PA-2A, PA-2B, PA-3A and PA-3B using Nextera XT DNA kit. Libraries were sequenced using Illumina Miseq platform in pair-end mode, read length of 150 bp. Quality of the reads was checked using FastQC version 0.11.8, standard settings. Reads were mapped to the reference genome of *L. monocytogenes* EGD-e (ASM19603v1, NCBI) using Breseq version 0.33.0 pipeline, standard settings (Deatherage and Barrick, 2014). SNPs were found using gdtools, and Single nucleotide polymorphisms (SNPs) present in both read directions for the isolates are listed in Table 1.

**Table 1:** Mutations found in PA tolerant strains based on whole genome sequencing.

PA tolerant strain	Location	Gene	Codon	Mutation	Mutation ID	Description
PA-1A PA-1B	1,111,021	*lmo1079*	85	Q➔STOP (CAA➔TAA)	*lmo1079-*sub1	A nonsense mutation, which results in the introduction of a STOP codon at codon 85 in *lmo1079.*
PA-2A PA-2B	1,113,036	*lmo1079*	756	Y➔STOP (TAC➔TAA)	*lmo1079*-sub2	A nonsense mutation, which results in the introduction of a STOP codon at codon 756 in *lmo1079.*
PA-1A PA-1B	1,117,421	*lmo1083*	110	∆1 bp (AAT➔ATG)	*lmo1083-*sub	A 1 bp deletion, which results in a frameshift mutation in *lmo1083*. The frameshift introduces a STOP codon at codon 115.
PA-3A PA-3B	1,928,124	*lmo1851*	101	G➔D (GGT➔GAT)	*lmo1851*-sub	A missense mutation, which results in amino acid substitution of codon 101, from glycine to aspartic acid, in *lmo1851.*

### Fluorescent Microscopy With Wheat Germ Agglutinin, Alexa Fluor™ 594 Conjugate

One milliliter of ON culture was centrifuged at 3,500 rpm for 3 min, and the supernatant was removed and resuspended in 100 μl 1x PBST (1x PBS + 0.1% Tween 20; Sigma-Aldrich). The resuspended cells were incubated for 5 min at room temperature (RT) in darkness with 50 μl 0.1 mg/ml Wheat Germ Agglutinin, Alexa Fluor™ 594 Conjugate (Invitrogen; resuspended in 1x PBS). The samples were centrifuged again and washed two times in 500 μl 1x PBST. The bacteria were resuspended in 1x PBST and spotted on poly-lysin [poly-lysin solution 0.1% (w/v); Sigma-Aldrich] coated microscopy slides. Bacteria were visualized by phase contrast (PH) and Texas-Red using an inverted fluorescence Olympus IX83 microscope. Images were analyzed using FIJI ImageJ (Schindelin et al., 2012).

### Hydrophobicity Assay

Overnight (ON) cultures (5 ml) were centrifuged for 5 min at 4,000 rpm, the supernatant was removed, and pellet was washed three times in 5 ml 1x PBS. Pellet was resuspended in 1x PBS and diluted to OD_600_ = 0.3 (OD_600__1). Three milliiliter of the diluted cultures was transferred to culture tubes and 300 μl n-hexadecane (Sigma-Aldrich) was added. Samples were vortexed for 2 min followed by incubation for 15 min at RT for phase separation. OD_600_ was measured again for the water phase (OD_600__2), and the percentage of cells staying in the hydrophilic phase was calculated by OD_600__2/OD_600__1 x 100%. Data were analyzed by one-way ANOVA with Bonferroni’s multiple-comparison test. Only differences with at least 95% CI were reported as statistically significant.

### Membrane Potential

Membrane potential was measured using BacLight Bacterial Membrane Potential Kit (Invitrogen). ON cultures were diluted to OD_600_ = 0.003 in 1 x PBS and split into falcon tubes (1 ml in each). Ten microliter of a proton ionophore carbonyl cyanide 3-chlorophenylhydrazone (CCCP) 500 μM was added to the depolarization control and incubated for 5 min. Ten microliter of the fluorescent membrane-potential indicator dye 3,3′-diethyloxa-carbocyanine iodide (DiOC_2_) was then added to the samples and the samples were incubated in darkness for 15–30 min followed by fluorescence-activated cell sorting using FACSAria II. One hundred thousand events were measured for each sample.

### BODIPY-C12 Binding Assay

Overnight (ON) cultures (5 ml) were centrifuged at 3,500 rpm for 5 min. The supernatant was removed, and pellet was washed twice in 5 ml 1x PBS. After the washing step, bacteria were resuspended in 1x PBS and OD_600_ was measured. Samples were diluted in 1x PBS to OD_600_ = 0.3. Each sample was split into two falcon tubes (1 ml in each), one was incubated with 5 μl of vehicle (nonfluorescent) and the other with 5 μl of 0.5 mg/ml BODIPY™ 558/568 C_12_ (Invitrogen; diluted in 96% ethanol) for 5 min at 4°C. The fluorescence of the samples was measured using FACSAria II, with the following parameters: 100,000 events per sample, neutral density filter size 0.5 and a flow rate of 1. The mean background fluorescence of each sample (vehicle) was subtracted from the mean fluorescence of the BODIPY treated samples, before ratios were calculated. Data were analyzed by one-way ANOVA with Bonferroni’s multiple-comparison test. Only differences with at least 95% CI were reported as statistically significant.

## Results

### Selection of PA Tolerant Mutants

**Figure 1:**
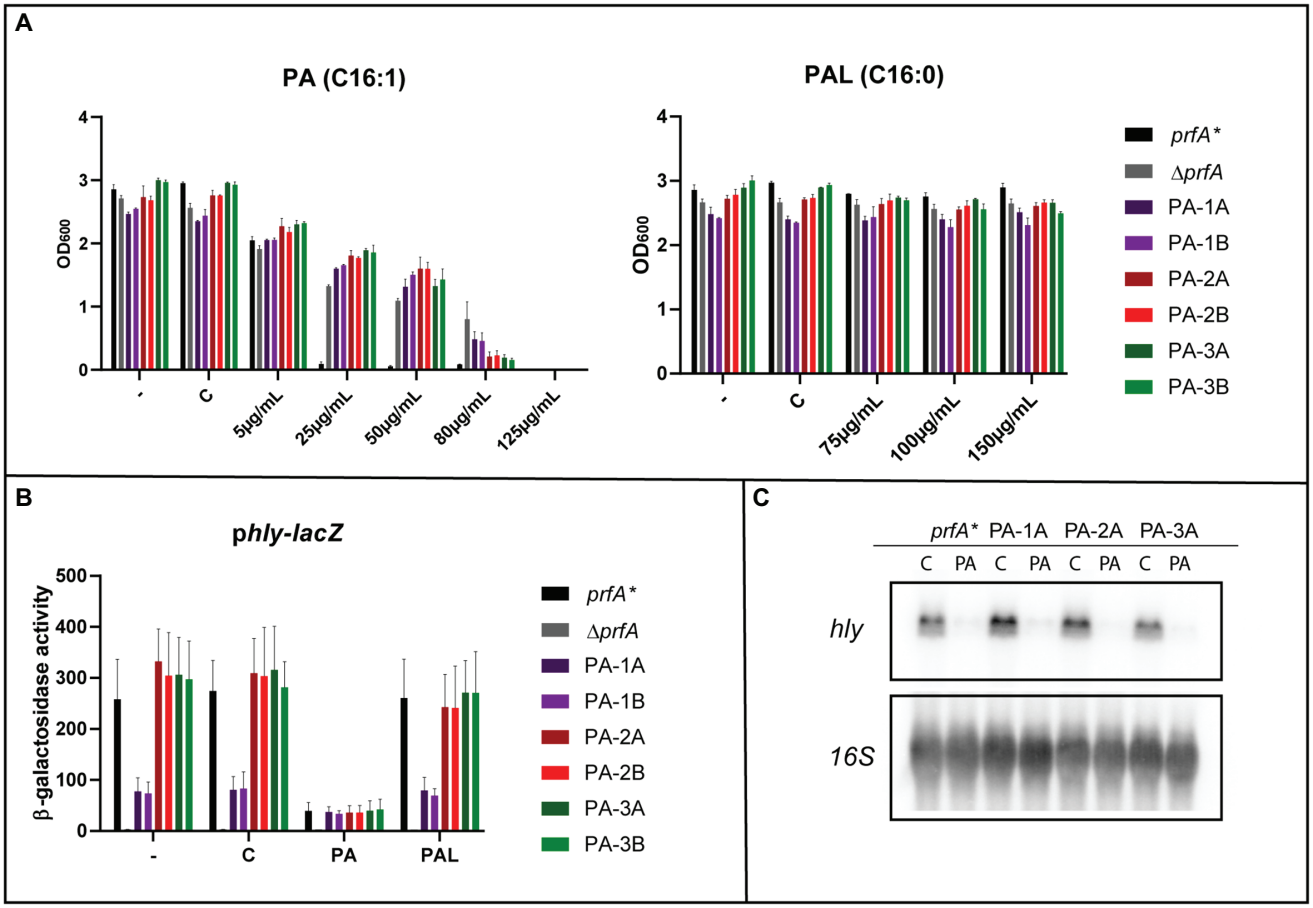
The response of selected palmitoleic acid (PA) tolerant isolates toward free fatty acid (FFA) exposure. **(A)** Growth of prfA*, ∆*prfA* and the six selected PA tolerant strains upon exposure to different concentrations of PA or palmitic acid (PAL). As controls, cultures were left untreated (−) or exposed to vehicle (C). OD_600_ values were measured after 20 h of growth. Results are the average of three independent experiments. **(B)** Expression of the PrfA-dependent virulence gene *hly* upon exposure to PA or PAL. The promotor region of *hly* cloned into the reporter plasmid pTCV-*lacZ* was transformed into prfA*, ∆*prfA* and the six selected PA mutants. The resulting strains were grown to OD_600_ = 0.3 and exposed to 2 μg/ml PA or 150 μg/ml PAL. As controls cells were left untreated (−) or incubated with a concentration of vehicle (C) corresponding to the one used for FFA conditions. Samples were harvested after 20 h and used for β-galactosidase assays. Results are the average of three independent experiments, each performed in technical duplicates. **(C)** Northern blot analysis of *hly* mRNA levels upon exposure to PA. The prfA* strain and three selected PA mutants were grown to OD_600_ = 0.3 and exposed to 2 μg/ml PA or as control a corresponding concentration of vehicle (C). Cells were harvested after 1 h. Northern blots were probed for *hly* mRNA and 16S rRNA (loading control). The experiment was performed in three biological replicates.

FFA tolerant strains were isolated in a prfA* background to allow further investigation of whether the mutations obtained confer tolerance toward the antimicrobial activity as well as the PrfA-inhibitory effect of PA (Sternkopf Lillebæk et al., 2017; Dos Santos et al., 2020). To select for PA tolerance, the prfA* strain was diluted in BHI and incubated overnight (ON) with PA; the concentration of PA was increased by 2-fold each day. A total of six PA tolerant candidates were isolated from three independent cultures after growth in BHI with 125 μg/ml PA. To investigate their PA tolerance, the six isolates were cultured ON with increasing concentrations of PA (Figure 1A). As controls, the parental strain (prfA*) and a PrfA-deficient strain (∆*prfA*) were included; ∆*prfA* is known to exert increased tolerance toward FFAs (Sternkopf Lillebæk et al., 2017; Dos Santos et al., 2020). As shown in Figure 1A, growth of prfA* was restricted at 25 μg/ml of PA, whereas growth of the six isolates and ∆*prfA* was prevented at 125 μg/ml of PA, demonstrating that the isolated strains are 5-fold more tolerant against PA compared to the parental strain. Additionally, growth in presence of PA’s saturated counter partner, the non-antimicrobial FFA palmitic acid (PAL, C16:0), was evaluated (Figure 1A). This growth experiment revealed that all six isolates, as well as the two control strains, could grow in presence of PAL. Altogether, the six isolated strains exhibit tolerance toward PA, whereas their growth is unaffected by PAL.

### PA Tolerant Strains Are Still Sensitive Toward the PrfA Inhibitory Effect of FFAs

Since PA is also known to inhibit the activity of PrfA (Sternkopf Lillebæk et al., 2017), we performed a β-galactosidase assay to investigate if the six PA tolerant strains had become insensitive toward the PrfA inhibitory effect of FFAs. The six strains were transformed with the p*hly-lacZ* fusion plasmid, which contains the PrfA-dependent promotor region of *hly* fused to *lacZ*. The prfA* and ∆*prfA* strains were again included as controls. The resulting eight strains were grown to mid-exponential phase; then, the cultures were split and treated with sub-inhibitory levels of PA or PAL. Untreated and vehicle treated cultures were included as controls. After 20 h of growth, cells were harvested; the results from the β-galactosidase assay are presented in Figure 1B. Except for ∆*prfA*, the β-galactosidase activity ranged from 80 to 300 units for all strains tested during regular growth and after exposure to the vehicle or the non-inhibitory FFA PAL. These results demonstrate that all six PA tolerant mutants encode a functional PrfA protein. Upon exposure to sub-inhibitory levels of PA, the β-galactosidase activity was remarkably reduced for prfA* and the six isolated mutants. These data show that the PA tolerant isolates are still sensitive toward the PrfA inhibitory activity of PA. Notably, high levels of β-galactosidase activity were observed for all strains containing the PrfA independent p*lhrA36-lacZ* fusion plasmid under all growth conditions tested (Supplementary Figure S1).

In addition to the β-galactosidase assays, northern blotting was performed to evaluate the effect of PA on the expression of *hly*. Three PA tolerant strains and prfA* were grown to mid-exponential phase and exposed to a sub-inhibitory level of PA for 1 h. Results from the northern blot are shown in Figure 1C. The mRNA level of *hly* was strongly reduced in all four strains upon exposure to PA, indicating that PA acts to inhibit PrfA dependent virulence gene expression in the PA tolerant strains. When comparing the results of the β-galactosidase assay and the northern blot analysis under control conditions, we note that *hly-lacZ* expression in isolates PA-1A and PA-1B is lower relative to the other strains, whereas *hly* mRNA levels are comparable for all strains tested (Figures 1B,C). Most likely, these isolates are slightly more resistant to the conditions used for cell lysis in β-galactosidase assays versus northern blotting. In line with this, the PA dependent increase in *lhrA36-lacZ* expression could be due to an increased susceptibility of PA-1A/1B to cell lysis after PA exposure (Supplementary Figure S1).

Altogether, the results from the β-galactosidase assay and the northern blot experiment confirm that any kind of mutations, which might have occurred in the PA tolerant strains, are only relevant to studies of the antimicrobial activity of FFAs.

### GlcNAc Glycosylation on the WTAs Contributes to PA Sensitivity in *Listeria monocytogenes*

To reveal the type of mutations that led to PA tolerance, the six isolates and the parental strain prfA* were whole genome sequenced (WGS). As shown in Tables 1, a total of four mutations were found in the PA tolerant strains. Isolates from the first biological replicate carry two mutations: a frameshift mutation in *lmo1083* and a nonsense mutation in *lmo1079*. The two isolates from the second biological replicate carry a nonsense mutation in *lmo1079* as well, whereas isolates from the third biological replicate carry a missense mutation in *lmo1851*. The latter gene (*lmo1851*) encodes for a carboxyl-terminal protease, whereas both *lmo1079* and *lmo1083* encode enzymes involved in modification of WTAs in *L. monocytogenes* serovar 1/2a. More specifically, *lmo1079* encodes an YfhO homolog that is essential for the GlcNAc glycosylation of WTAs (Rismondo et al., 2018), whereas *lmo1083* (*rmlB*) is a part of the *rmlACBD* locus encoding the RmlABCD proteins that are essential for rhamnosylation of WTAs (Carvalho et al., 2015). We assume that the two different nonsense mutations found in *lmo1079* will result in truncated Lmo1079 protein variants, due to premature termination of translation. The frameshift mutation found in *lmo1083* leads to the formation of a STOP codon at codon 115 out of 329, which is expected to cause loss of function and result in lack of rhamnosylation, as seen for similar mutations studied previously (Denes et al., 2015; Eugster et al., 2015) Since three of the four detected mutations are present in genes encoding for WTA glycosylation enzymes, we decided to focus on the role of *lmo1079* and *lmo1083* in the response toward antimicrobial FFAs.

**Figure 2:**
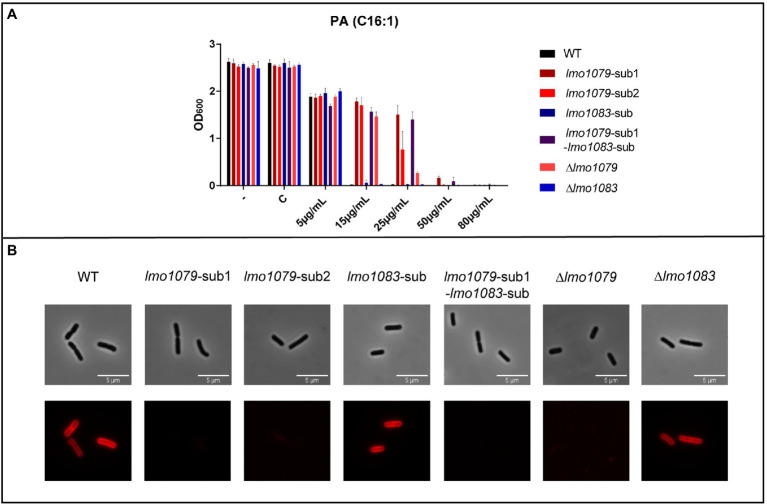
The impact of *lmo1079* and *lmo1083* mutations on PA tolerance and GlcNAc glycosylation of wall teichoic acid (WTA). **(A)** Growth of wild-type and *lmo1079* and *lmo1083* mutant strains upon PA exposure. Strains were diluted to OD_600_ = 0.0002 and exposed to various concentrations of PA. As controls cultures were left untreated (−) or exposed to the vehicle (C). Results are the average of three independent experiments. **(B)** Detection of GlcNAc on WTAs. Wild-type, *lmo1079* and *lmo1083* mutant cells were harvested and resuspended in 1x PBST. Alexa Fluor™ 594 conjugated WGA was added to a final concentration of 33 μg/ml. Samples were washed and resuspended in 1 x PBST and cells were visualized on poly-lysin coated slides by fluorescence microscopy. The experiment was performed in triplicates; one representative example is shown.

To investigate if mutations in *lmo1079* and *lmo1083* contribute to PA tolerance, the three single mutations *lmo1079* [Q85 (STOP)), *lmo1079* (Y756 (STOP)] and *lmo1083* (position 329 nt, ∆1 bp), from now on referred to as *lmo1079*-sub1, *lmo1079*-sub2 and *lmo1083*-sub, respectively, were introduced into the wild-type strain. Additionally, we constructed a double mutant harboring both *lmo1079*-sub1 and *lmo1083*-sub. Furthermore, since all three mutations are expected to affect the functionality of the resulting proteins, deletion mutants for *lmo1079* and *lmo1083* were constructed as controls. Initially, the mutants and wild-type were cultured in BHI medium (Supplementary Figure S2), which showed that none of the mutations affect bacterial growth. To examine if the mutations lead to PA tolerance, growth was studied upon exposure to increasing concentrations of PA (Figure 2A). Growth of the wild-type, *lmo1083*-sub and Δ*lmo1083* mutant strains was prevented at 15 μg/ml PA, whereas growth of the *lmo1079* mutants was restricted at higher concentrations of PA (50 or 80 μg/ml). Thus, mutations in *lmo1079* confer tolerance toward PA by 3–5-fold compared to the wild-type strain. To confirm that the *lmo1079* mutations are causing PA tolerance, growth experiments with increasing concentrations of PA were performed for complementation mutants as well (Supplementary Figure S3A). The *lmo1079*-sub1 mutation was complemented on the chromosome of the single and double mutant, referred to as *lmo1079*-sub1::*lmo1079*-c and *lmo1079*-sub1-*lmo1083*-sub::*lmo1079*-c, respectively. In both cases, complementation of the *lmo1079*-sub1 mutation restores the phenotype back to wild-type, which confirms that mutation of *lmo1079* confers PA tolerance.

To address if the PA tolerance can be linked to GlcNAc glycosylation of WTAs, we studied the presence of GlcNAc on the WTAs of wild-type and various *lmo1079* and *lmo1083* mutant strains, using fluorescence microscopy to detect fluorescently labeled Wheat Germ Agglutinin (WGA) selective for GlcNAc (Figure 2B). Wild-type and *lmo1083* single mutants fluoresce, confirming that they have GlcNAc expressed on the WTAs, whereas no signal was seen for all *lmo1079* mutants tested. Based on these observations, we conclude that the mutants, which had acquired tolerance toward PA, are lacking the GlcNAc glycosylation on their WTAs.

### Lack of GlcNAc on WTAs Induces Tolerance Toward Multiple Antimicrobial FFAs

**Figure 3:**
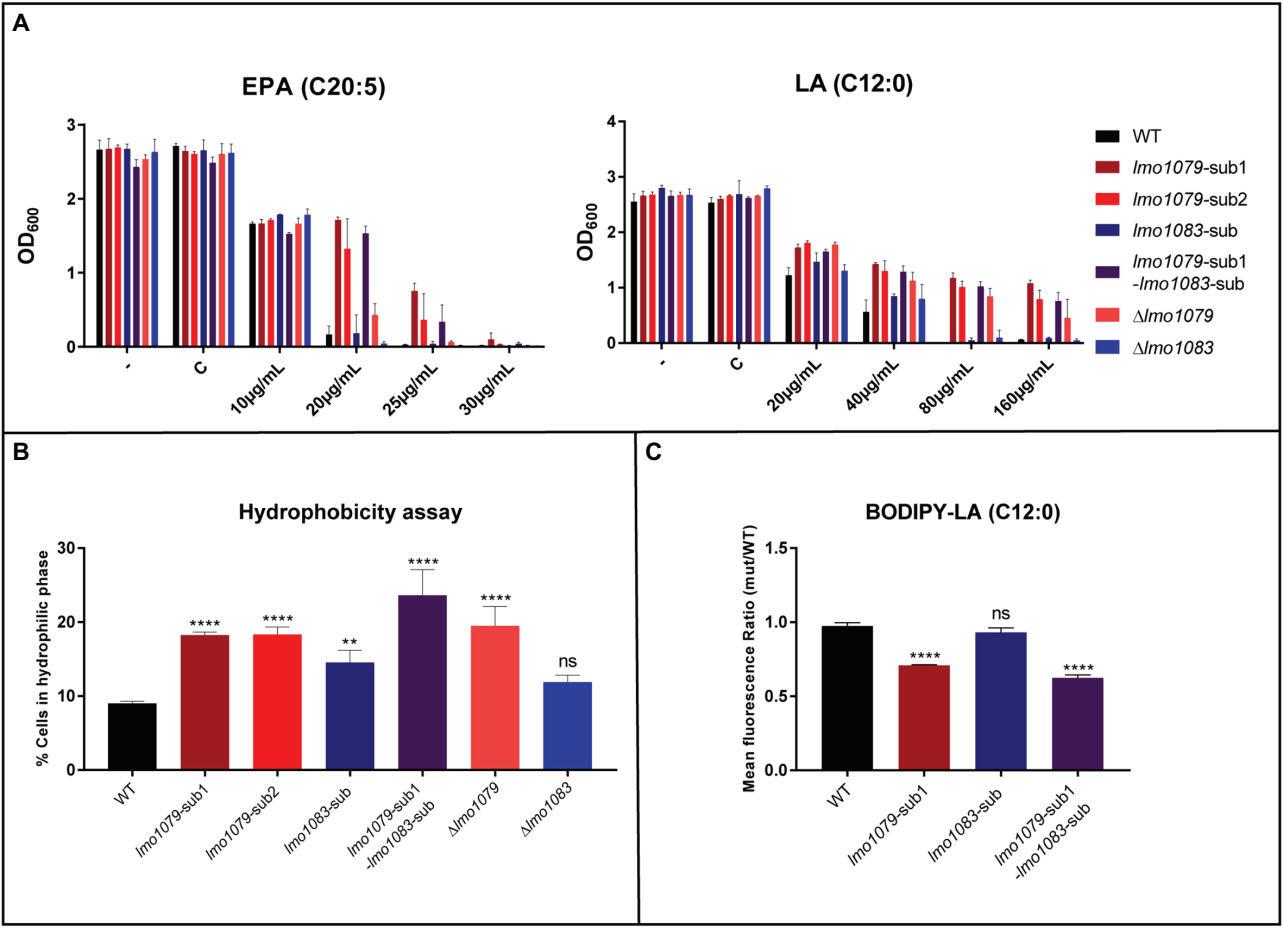
The role of WTA modifications in FFA tolerance. **(A)** Wild-type, *lmo1079* and *lmo1083* mutant strains were diluted to OD_600_ = 0.0002 and exposed to different concentrations of eicosapentaenoic acid (EPA) or LA. As controls, cultures were left untreated (−) or exposed to the vehicle (C). Growth was measured after 20 h. Results are the average of three independent experiments. **(B)** Hydrophobicity of the bacterial surface. Bacteria from overnight (ON) cultures were harvested and washed in 1x PBS. Cells were resuspended in 1x PBS to OD_600_ = 0.3 and vortexed together with *n-*hexadecane. OD_600_ was measured again for the hydrophilic phase after phase separation. Percentage of cells in hydrophilic phase was determined. Results are the average of three independent experiments. Statistical analysis was performed by one-way ANOVA with Bonferroni’s multiple-comparison test, (ns) = not significant, (**) = *p* < 0.01, (****) = *p* < 0.0001. **(C)** LA binding assay. Bacteria were harvested and washed in 1x PBS. Cells were diluted in 1x PBS to OD_600_ = 0.3 and incubated with 2.5 μg/ml BODIPY-C12 or a corresponding concentration of vehicle as control. Bacterial fluorescence was determined by fluorescence-activated cell sorting (FACS). Results are mean fluorescence relative to wild-type and the average of three independent experiments. Statistical analysis was performed by one-way ANOVA with Bonferroni’s multiple-comparison test, (ns) = not significant, (****) = *p* < 0.0001.

Since multiple FFAs have shown antimicrobial properties against *L. monocytogenes* (Sternkopf Lillebæk et al., 2017; Dos Santos et al., 2020), we wanted to study if the absence of GlcNAc on WTAs results in increased tolerance toward other antimicrobial FFAs than PA. Strains were grown in increasing concentrations of the long-chain polyunsaturated FFA eicosapentaenoic acid (EPA, C20:5) and the medium-chain saturated FFA lauric acid (LA, C12:0). The results from the growth experiments are presented in Figure 3A. In presence of EPA, *lmo1079* mutants show increased tolerance compared to the wild-type and *lmo1083* mutants. The *lmo1079* mutants are, however, still quite sensitive toward the presence of EPA. For LA, the same tendency is observed as for PA in Figure 2A, since the growth of EGD and *lmo1083* mutants is inhibited at 80 μg/ml of LA, while all *lmo1079* mutants are still capable of growing at 160 μg/ml of LA. Lack of GlcNAc clearly induces tolerance toward LA and, to a lesser extent, EPA. Based on these findings, we conclude that if GlcNAc is absent on the WTAs, *L. monocytogenes* becomes more tolerant toward different types of antimicrobial FFAs.

### Changes in WTA Glycosylation Result in a More Hydrophilic Bacterial Surface

In *Staphylococcus aureus*, it is known that WTAs are important for maintaining tolerance toward antimicrobial FFAs. More specifically, in the absence of WTAs the bacterial surface becomes more hydrophobic and increases the sensitivity of *S. aureus* toward antimicrobial FFAs (Kohler et al., 2009). Our data show that lack of the GlcNAc glycosylation on WTAs in *L. monocytogenes* causes FFA tolerance. Therefore, we investigated if changes in WTA decorations affect the hydrophobicity of the bacterial surface in *L. monocytogenes* by studying the microbial adhesion to *n*-hexadecane using the hydrocarbon test (Rosenberg et al., 1980; Kohler et al., 2009). The percentage of bacteria that stayed in the hydrophilic phase is presented in Figure 3B. For all *lmo1079* mutants, a higher percentage of the cells stayed in the hydrophilic phase, relative to the wild-type, whereas *lmo1083* mutants were only slightly (*lmo1083*-sub) or not significantly different (Δ*lmo1083*) from wild-type. The increased FFA tolerance, which was observed for the *lmo1079* mutants, thereby correlates with a more hydrophilic surface. Furthermore, by complementing *lmo1079-*sub1 the hydrophobicity of the bacterial surface was restored to the level of the parental strain (Supplementary Figure S3B). Finally, we examined if *lmo1079*-sub1 and/or *lmo1083*-sub affect the membrane potential of *L. monocytogenes* compared to the parental strain. The results obtained confirmed that none of the mutations had a detectable effect on the membrane potential (Supplementary Figure S4). Based on these observations, we conclude that lack of GlcNAc entails a more hydrophilic bacterial surface in *L. monocytogenes* resulting in an increased tolerance to FFA.

### Antimicrobial FFAs Are Repulsed From the Surface of *Listeria monocytogenes* in Absence of GlcNAc on the WTAs

Since *lmo1079* mutants have a more hydrophilic surface, it could be speculated that FFAs are repulsed from the cell surface when GlcNAc is lacking. This hypothesis was tested by measuring the interaction between fluorescent-labeled LA (BODIPY FFA C12) and the bacterial surface by fluorescence-activated cell sorting (FACS). The mean fluorescence of single and double mutants relative to the parental strain is presented in Figure 3C. The mean fluorescence of the *lmo1083* single mutant is comparable to the parental strain, indicating that the FFA interacts with the bacterial surface to the same extent regardless of the mutation in the rhamnosylation gene. However, a significant decrease in the mean fluorescence was observed when *lmo1079* is mutated, showing that the FFA cannot efficiently interact with the bacterial surface, when GlcNAc modification of WTA is absent. FFAs are therefore most likely repulsed from the bacterial surface when GlcNAc is missing on the WTAs of *L. monocytogenes*.

### GlcNAc Modification of WTA Mediates Sensitivity Toward Acid Stress

**Figure 4:**
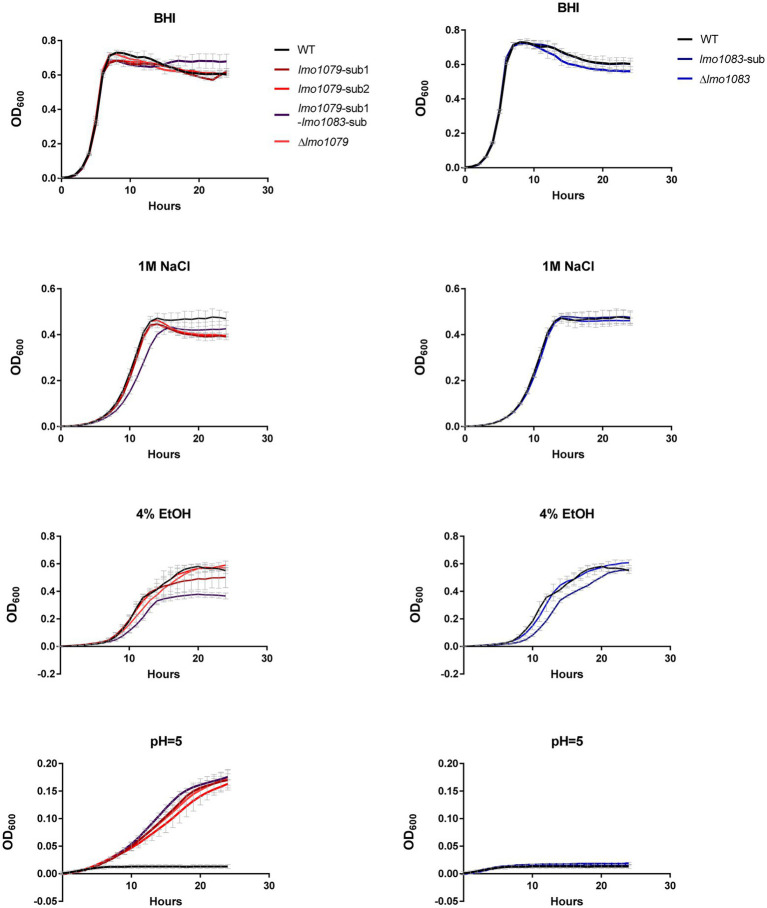
Growth of WTA modification mutants upon exposure to different stress conditions. Strains were diluted to OD_600_ = 0.005 in 200 μl growth medium in 96-well plates. The growth medium employed was either untreated BHI medium (BHI), BHI supplemented with 4% ethanol (4% EtOH), 1 M NaCl, or adjusted to pH = 5. Cultures were incubated for 24 h and growth was measured regularly by a microplate reader. Results are the average of three independent experiments.

WTA decorations have previously been shown to be essential for *L. monocytogenes*’ ability to invade host cells (Sumrall et al., 2020), anchoring membrane proteins (Sumrall et al., 2020) and maintaining resistance toward antimicrobial peptides and sensitivity toward antibiotics (Carvalho et al., 2015; Meireles et al., 2020). Therefore, we examined if the PA tolerant mutants were affected in their tolerance toward other environmental stress conditions, which *L. monocytogenes* may encounter upon disinfection, host infection, food production and preservation. The *lmo1079* and *lmo1083* mutants were, together with the parental strain, grown in 96-well plates under different stress conditions, e.g., salt, ethanol, and acid. The results from the growth experiments are presented in Figure 4. All strains grew equally well in BHI medium. In the presence of salt or ethanol, growth of the double mutant was slightly impaired relative to the single mutants and wild-type. Upon exposure to acidic conditions, a clear growth phenotype was observed for the *lmo1079* mutants; they were all able to grow at pH = 5, whereas growth of wild-type and the *lmo1083* single mutants was restricted. The acid tolerant phenotype was confirmed to be strictly dependent on the lack of GlcNAc, as complementation of *lmo1079*-sub1 restores wild-type sensitivity (Supplementary Figure S5). Altogether, mutations of both WTA substitution genes caused a slight growth deficiency upon exposure to salt and ethanol. Notably, lack of GlcNAc on WTAs not only resulted in FFA tolerance, but also tolerance toward acid stress. Altogether, our findings demonstrate that GlcNAc modification of WTA plays a role in the response toward stress conditions encountered by *L. monocytogenes* during life as a saprophyte and a pathogen.

## Discussion

Microbial development of resistance toward traditional antibiotics is expanding worldwide much faster than new antibiotics are discovered (Ventola, 2015). Due to the rapid increase in antibiotic resistance, alternative treatment options and combination therapies are attracting more attention (Brown, 2015). Natural compounds like FFAs are known to have antimicrobial properties against pathogens, however, the specific mechanism of action is not fully understood (Desbois and Smith, 2010). In fact, multiple organisms (plants, mice, humans, etc.) use antimicrobial FFAs as a natural protection mechanism to avoid bacterial infection (Kengmo Tchoupa et al., 2021). Pathogenic bacteria therefore use different strategies to avoid FFA toxicity, so they can adapt to the host environment and cause infections despite the presence of host delivered antimicrobial FFAs. These bacterial defense strategies may involve changes in surface polarity, export of FFAs or detoxification of antimicrobial FFAs (Kengmo Tchoupa et al., 2021). In *S. aureus*, the FFAs are repulsed from the bacterial surface by different mechanisms. *Staphylococcus aureus* induces the expression of capsule biosynthesis genes upon exposure to FFAs, as the polysaccharides in the capsule make the bacterial surface more hydrophilic (Mortensen and Kapral, 1992; Kenny et al., 2009; Moran et al., 2017). Furthermore, WTAs on the surface of *S. aureus* are known to be hydrophilic and hinder the interaction between the FFAs and bacterial membrane (Kohler et al., 2009). Additionally, *S. aureus* expresses the cell-wall anchored protein iron-regulated surface determinant A (IsdA), which decreases the surface hydrophobicity and hamper binding of the FFAs to the bacterium (Clarke et al., 2007). All these hydrophilic factors will naturally increase tolerance toward antimicrobial FFAs in *S. aureus* by repulsing the host delivered FFAs from the bacterial surface. If the intracellular concentration of antimicrobial FFAs in the bacterial cytosol exceed the inhibitory threshold, detoxification can occur by efflux systems, such as the resistance-nodulation-cell division (RND) superfamily (Kengmo Tchoupa et al., 2021). The FFAs are then exported out of the bacterium into the extracellular environment to reduce the intracellular concentration and promote bacterial growth (Alnaseri et al., 2015; Kengmo Tchoupa et al., 2021). Alternatively, FFAs can be detoxified by either oleate dehydratases or fatty-acid-modifying enzyme (FAME) after uptake to prevent intracellular accumulation (Long et al., 1992; Mortensen and Kapral, 1992; Volkov et al., 2010; Subramanian et al., 2019). The antimicrobial FFAs are metabolized into hydroxy-FFAs or esterified FFAs, which are less toxic and will promote cell survival (Long et al., 1992; Subramanian et al., 2019). Pathogens thereby possess multiple natural protection strategies against antimicrobial FFAs.

To examine how *L. monocytogenes* responds to antimicrobial FFAs, we studied mutations occurring in selected PA tolerant strains. Most of the isolated PA tolerant strains carry mutations in genes encoding for enzymes essential for WTA substitutions; *lmo1079* and *lmo1083* (*rmlB*; Table 1). The glycosyltransferase (Lmo1079) is responsible for transferring GlcNAc from a lipid carrier molecule in the outer leaflet of the bacterial membrane onto the growing ribitol backbone of Type I WTAs (Eugster et al., 2015; Rismondo et al., 2018). RmlB on the other hand is involved in the dTDP-L-Rha biosynthetic pathway, which is essential for construction of dTDP-L-Rha for the Type I WTA rhamnosylation (Carvalho et al., 2015). Since both genes are involved in WTA glycosylations, it could be speculated that the mutations in these two genes contribute to PA tolerance. However, our studies confirmed that only mutations in *lmo1079* induced PA tolerance, whereas the effect of mutating *lmo1083* was insignificant. This also might explain why no single *lmo1083* mutants were identified among the PA tolerant isolates. Notably, there seems to be a competition between Rhamnose and GlcNAc modification of WTA at 30 and 37°C (Tokman et al., 2016). In the present study, the PA tolerant strains were selected and analyzed at 37°C. Future studies should focus on testing the putative role of WTA decorations in FFA tolerance at lower temperatures.

Mutation of *lmo1079* induced tolerance toward different types of antimicrobial FFAs (saturated, mono- and poly-unsaturated), demonstrating that *lmo1079* plays an important role in the response of *L. monocytogenes* to antimicrobial FFAs in general. The acquired FFA tolerance of the *lmo1079* mutant strains correlated with a common absence of GlcNAc on their WTAs (Figure 2B), suggesting that lack of GlcNAc on Type I WTAs of *L. monocytogenes* confers FFA tolerance. As mentioned above, the presence of WTAs on *S. aureus* is necessary to sustain tolerance toward exogenous FFAs (Kohler et al., 2009). Thus, WTAs in general repulse FFAs from the bacterial surface and induce tolerance, whereas the GlcNAc modification seems to cause sensitivity, as lack of GlcNAc contribute to FFA tolerance. In *L. monocytogenes*, the GlcNAc modification thereby plays a separate role in relation to the response to antimicrobial FFAs, which is opposite to the role shown for WTAs in general in *S. aureus*.

The absence of WTAs in *S. aureus* causes a more hydrophobic bacterial surface; the FFAs are then more efficiently drawn to the surface, which increases sensitivity to FFAs (Kohler et al., 2009). These observations made us to speculate if WTA glycosylations in *L. monocytogenes* affect surface polarity as well. Our data show that lack of GlcNAc on the WTAs, through mutation or deletion of *lmo1079*, led to a more hydrophilic surface compared to *lmo1083* mutants and the parental strain (Figure 3B). These observations are further supported by data from (Brauge et al., 2018), where deletion of the two other genes involved in GlcNAc glycosylation (*lmo2549* and *lmo2550*) results in decreased hydrophobicity of the bacterial surface. The FFA tolerance caused by the absence of GlcNAc is therefore most likely due to a more hydrophilic surface. In support of this theory, we found that lack of GlcNAc resulted in reduced interaction between the antimicrobial FFA, LA, and the bacterial surface (Figure 3C). Thus, the FFAs are repulsed from the bacterial surface when GlcNAc is lacking, due to a more hydrophilic surface. Altogether, our observations support that the WTA decoration GlcNAc interferes with the surface polarity and thereby the ability of FFAs to interact with the bacterial surface.

WTA glycosylations of *L. monocytogenes* serovar 1/2a have shown to be crucial for sustaining tolerance toward other antimicrobial compounds. For example, absence of GlcNAc slightly increases the sensitivity toward the antimicrobial peptide CRAMP (Meireles et al., 2020). Notably, the sensitivity is amplified if both GlcNAc and Rha are lacking on the WTAs, indicating that the two types of WTA decorations cooperate to confer resistance toward antimicrobial peptides (Meireles et al., 2020). In addition, both WTA glycosylations confer decreased susceptibility of *L. monocytogenes* serovar 1/2a toward the antibiotics, gentamicin and ampicillin, which are commonly used for treatment of Listeriosis (Meireles et al., 2020). In our study, we furthermore found that lack of GlcNAc alone increases the tolerance of *L. monocytogenes* toward acid stress, and mutations of both *lmo1079* and *lmo1083* led to a slight increase in bacterial susceptibility toward ethanol and salt stress. Importantly, WTA glycosylations are known to serve as binding targets for bacteriophages in the environment and changes in these structures therefore prevent phage adsorption to WTAs (Denes et al., 2015; Eugster et al., 2015). Bacteriophage resistance for Type I WTA serovars is caused by point mutations in genes belonging to the *rmlACBD* operon (responsible for Rha modifications), or genes encoding for the GlcNAc glycosylation enzymes (i.e., *lmo1079*, *lmo2549* or *lmo2550*; Denes et al., 2015; Eugster et al., 2015). Altogether, these findings substantiate an important role for GlcNAc glycosylation of WTA, alone or together with Rha modifications, in the response of *L. monocytogenes* toward various antimicrobial agents, environmental stress conditions, and bacteriophages.

As mentioned above, the WTA glycosylation GlcNAc contributes to sensitivity toward antimicrobial agents, such as FFAs and bacteriophages, so why does *L. monocytogenes* retain the GlcNAc decorations? As previously described, GlcNAc contributes to tolerance toward antimicrobial peptides (Meireles et al., 2020). Additionally, the GlcNAc WTA substitution has been shown to be important for adhesion and biofilm formation (Brauge et al., 2018). The lack of GlcNAc results in decreased attachment of *L. monocytogenes* to surfaces and the biofilm architecture that arises is different compared to wild-type. The difference in biofilm structure is expected to be caused by a more hydrophilic bacterial surface (Brauge et al., 2018). Lack of GlcNAc results in a less efficient biofilm structure, which reduce adherence and increase sensitivity toward cleaning procedures (Brauge et al., 2018). Clearly, this outcome of lacking GlcNAc is therefore not beneficial for bacterial survival under these conditions. Additionally, it is known that the most glycosylated serovars of *L. monocytogenes* (serovar 1/2a, 1/2b, and 4b) represent the majority of outbreak strains worldwide (Maury et al., 2016; Braga et al., 2017; Datta and Burall, 2018; Smith et al., 2019), indicating that WTA decorations contribute to virulence. In line with this, change in serovar, caused by phage resistance, leads to virulence attenuation (Sumrall et al., 2020). To summarize, although GlcNAc decoration of WTA increases the sensitivity of *L. monocytogenes* toward some antimicrobial agents, the pathogen may retain GlcNAc on WTA to promote important functions related to biofilm formation, tolerance toward cleaning products and antimicrobial peptides, and possibly, to sustain virulence.

In conclusion, this work demonstrated for the first time, that the lack of GlcNAc on WTA protects *L. monocytogenes* against the antimicrobial activity of FFAs. We also showed that in the absence of GlcNAc, the FFAs are repulsed from the bacterium due to a more hydrophilic surface. Notably, the FFA PA efficiently inhibited the expression of PrfA-dependent virulence genes, irrespectively of the absence or presence of WTA decorations (Figures 1B,C). Thus, strains deficient of WTA decorations can still be targeted by the anti-virulence activity of FFAs.

## Data Availability Statement

The datasets presented in this study can be found in online repositories. The names of the repository/repositories and accession number(s) can be found at: https://www.ncbi.nlm.nih.gov/, PRJNA815746.

## Author Contributions

RT and BK contributed to the conception and design of the study and wrote the first draft of the manuscript. RT, PS, and ES contributed to the experimental work. RT, MS, and MK contributed to the sequencing analysis. All authors contributed to the article and approved the submitted version.

## Funding

This work was supported by the Novo Nordisk Foundation (grant number NNF17OC0028528).

## Conflict of Interest

The authors declare that the research was conducted in the absence of any commercial or financial relationships that could be construed as a potential conflict of interest.

## Publisher’s Note

All claims expressed in this article are solely those of the authors and do not necessarily represent those of their affiliated organizations, or those of the publisher, the editors and the reviewers. Any product that may be evaluated in this article, or claim that may be made by its manufacturer, is not guaranteed or endorsed by the publisher.
