# Supplementary material for: Absence of N-Acetylglucosamine Glycosylation on Listeria monocytogenes Wall Teichoic Acids Promotes Fatty Acid Tolerance by Repulsion From the Bacterial Surface
Source: Front Microbiol. 2022 May 12;13:897682. doi: 10.3389/fmicb.2022.897682 (PMC9133914; doi:10.3389/fmicb.2022.897682)
Supplement: Supplementary file 1 [file Data_Sheet_1.PDF]

Supplementary material

Absence of N-acetylglucosamine glycosylation on *Listeria monocytogenes* wall teichoic acids promotes fatty acid tolerance by repulsion from the bacterial surface

Rikke S. S. Thomasen[1], Patricia T. dos Santos[1,2], Eva M. Sternkopf Lillebæk[1], Marianne N. Skov[3], Michael Kemp[3,4] and Birgitte H. Kallipolitis[1]*

[1]Department of Biochemistry and Molecular Biology, University of Southern Denmark, Odense, Denmark.

[2]National Food Institute, Technical University of Denmark, Kgs. Lyngby, Denmark.

[3]Department of Clinical Microbiology, Odense University Hospital and Research Unit of Clinical Microbiology, University of Southern Denmark, Odense, Denmark.

[4]The Regional Department of Clinical Microbiology, Region Zealand, Zealand University Hospital, Koege, Denmark.

***Correspondence:** Birgitte H. Kallipolitis, bhk@bmb.sdu.dk

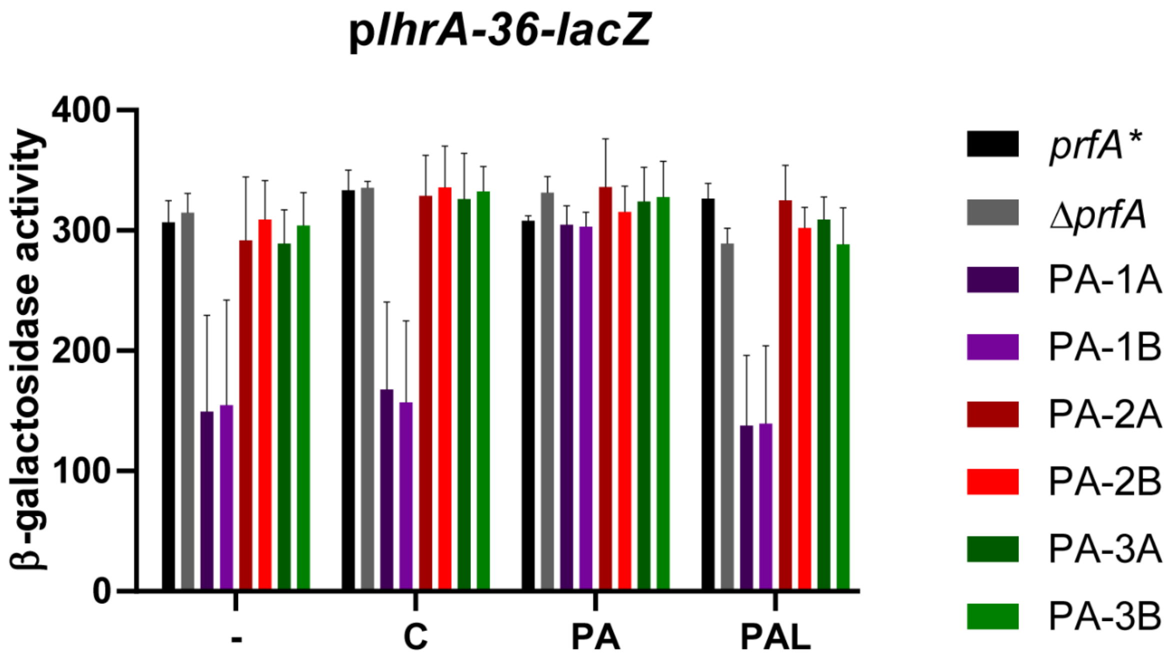

Suppl. Figure S1: Expression of p*lhrA-36-lacZ* in response to FFA exposure. The core promotor of *lhrA* cloned into the vector plasmid pTCV-*lacZ* was transformed into *prfA**, Δ*prfA* and the six PA tolerant strains. Resulting strains were grown to OD_{600}=0.3 and then exposed to either 2 µg/mL PA or 150 µg/mL PAL. Controls were left untreated (-) or exposed to the corresponding concentration of vehicle (C) as used for the FFA samples. After 20 h of growth cells were harvested. Results are the average of three independent experiments, each performed in technical duplicates.

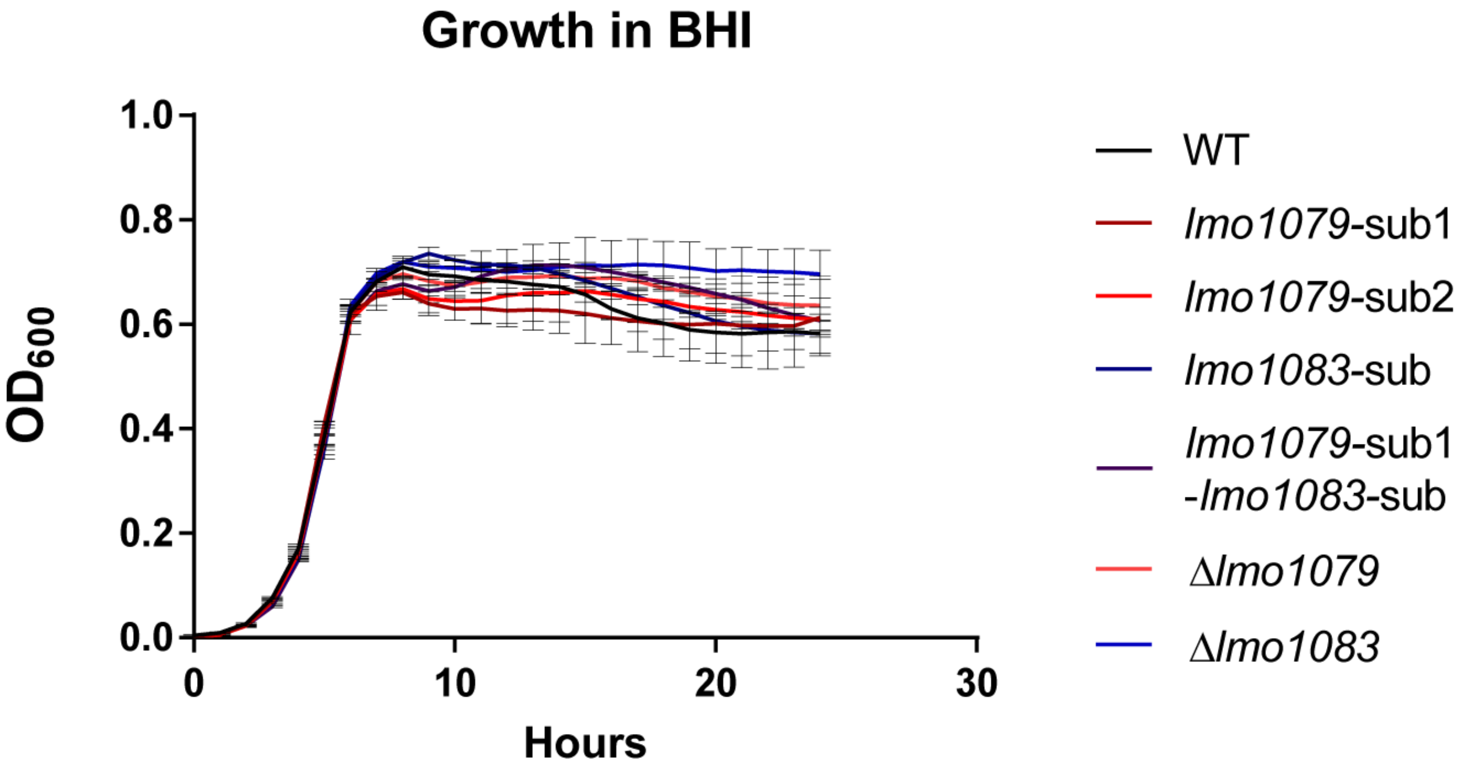

Suppl. Figure S2: Growth of WTA glycosylation mutants. Strains were diluted to OD_{600}=0.005 in a total volume of 200 µL in 96-well plates. Growth was measured regularly for 24 h by a microplate reader. Results are the average of three independent experiments.

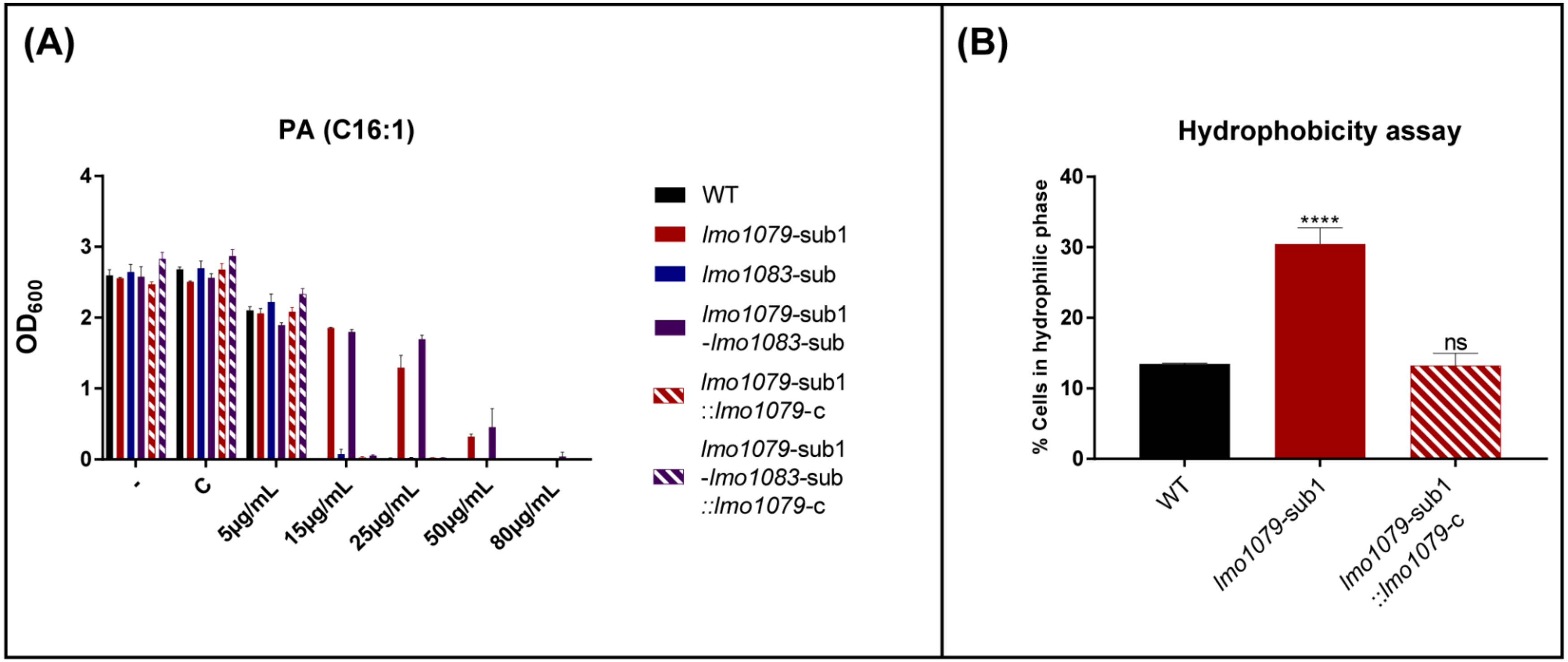

Suppl. Figure S3: Complementation of *lmo1079*-sub1. (**A**) Strains were diluted to OD_{600}=0.0002 and incubated for 20 h with different concentrations of PA. As controls, cultures were left untreated (-) or exposed to a corresponding concentration of vehicle as used in PA samples (C). Results are the average of three independent experiments. (**B**) Bacteria were harvested from ON cultures and washed in 1 x PBS. The cells were diluted in 1 x PBS to OD_{600}=0.3 and vortexed together with *n*-hexadecane. OD_{600} of the hydrophilic phase was measured again after phase separation. Percentage of bacteria in the hydrophilic phase was calculated. Results are the average of three independent experiments. Statistical analysis was performed by one-way ANOVA with Bonferroni's multiple-comparison test, (ns) = not significant, (****) = p<0.0001.

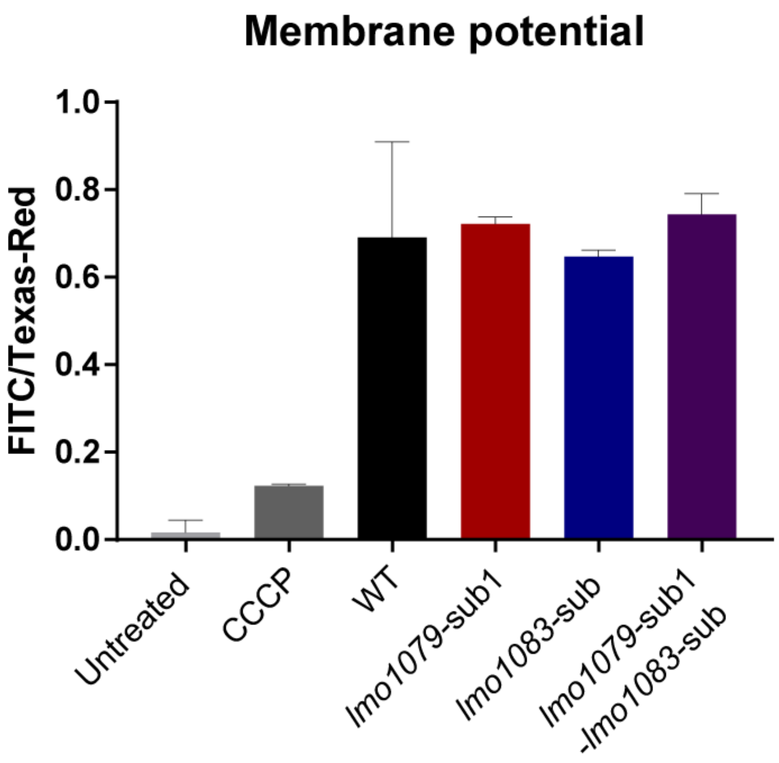

Suppl. Figure S4: Membrane potential of the WTA glycosylation mutants. Strains were diluted to OD_{600}=0.003 in 1 x PBS. As a depolarization control 5 µM CCCP was added to wild-type cells (CCCP) for 5 min. DiOC2 was added to the strains and the depolarization control, and the samples were incubated in darkness for 15-30 min followed by FACS. As control a wild-type culture was left untreated to measure the background fluorescence (Untreated). Results are the average of three independent experiments.

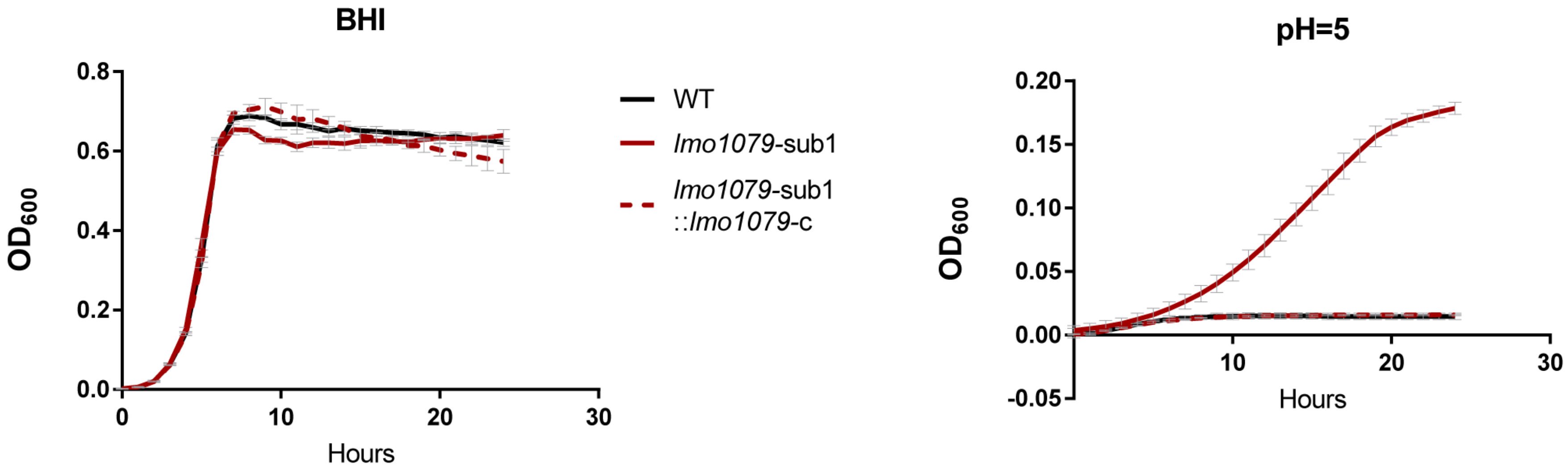

Suppl. Figure S5: Growth of *lmo1079*-sub1 mutant and complementation strains under acidic conditions. Strains were diluted to OD_{600}=0.005 in a total volume of 200 µL of regular BHI (BHI) or BHI pH adjusted to 5 with HCl (pH=5) in 96-well plates. Growth was measured for 24 h in microplate reader. Results are the average of three independent experiments.

Suppl. Table S1: Primers used in this study

Name	Sequence (5'→3')	Further information
**Cloning**		
P1 *lmo1079*-sub1	GGGG<u>GAATTC</u>TTGTACCAAGTCCTGCTGCCGGG	Forward primer for the upstream region of *lmo1079*-sub1. EcoRI restriction site is underlined.
P2 *lmo1079*-sub1	TCCCATTCCAATTT**A**CCAAGACCATAATTC	Reverse primer for the upstream region of *lmo1079*-sub1. The sub.1 substitution is presented in bold.
P3 *lmo1079*-sub1	GAATTATGGTCTTGG**T**AAATTGGAATGGGA	Forward primer for the downstream region of *lmo1079*-sub1. The sub1 substitution is presented in bold.
P4 *lmo1079*-sub1	GGGG<u>TCTAGA</u>CCAACAAATCACAAGAATTAACGGAACTGG	Reverse primer for the downstream region of *lmo1079*-sub1. XbaI restriction site is underlined.
P5 *lmo1079*-sub1	CAATTCCAGACGCAACTGC	Forward primer for chromosomal validation of *lmo1079*-sub1.
P6 *lmo1079*-sub1	CTTAGTAGCGGAATTCCTTGC	Reverse primer for chromosomal validation of *lmo1079*-sub1.
P1 *lmo1079*-sub2	GGGG<u>GAATTC</u>CCGCTTGAAAAAGAGGAAGCACTT	Forward primer for the upstream region of *lmo1079*-sub2. EcoRI restriction site is underlined.
P2 *lmo1079*-sub2	GGTAAGGTTGTTCCG**T**TAATAATTATAACGCAT	Reverse primer for the upstream region of *lmo1079*-sub2. The sub.2 substitution is presented in bold.
P3 *lmo1079*-sub2	ATGCGTTATAATTATTA**A**CGGAACAACCTTACC	Forward primer for the downstream region of *lmo1079*-sub2. The sub.2 substitution is presented in bold.

P4 *lmo1079*-sub2	GGGG<u>TCTAGA</u>TTCTGTTGATACTTCTTTTCCATCTAC	Reverse primer for the downstream region of *lmo1079*-sub2. XbaI restriction site is underlined.
P5 *lmo1079*-sub2	CCAGGCATTAGCCATACAGATG	Forward primer for chromosomal validation of *lmo1079*-sub2.
P6 *lmo1079*-sub2	GCTTTAAGCATTGGCGTTTC	Reverse primer for chromosomal validation of *lmo1079*-sub2.
P1 *lmo1083*-sub	GGGG<u>GAATTC</u>CGGACAAGGATACGAAACATCCACG	Forward primer for the upstream region of *lmo1083*-sub. EcoRI restriction site is underlined.
P2 *lmo1083*-sub	TTCTTTCGCAA**CAT**TAAAAGATTTAGCGTTCC	Reverse primer for the upstream region of *lmo1083*-sub. The substitution codon is presented in bold.
P3 *lmo1083*-sub	GGAACGCTAAATCTTTTA**ATG**TTGCGAAAGAA	Forward primer for the downstream region of *lmo1083*-sub. The substitution codon is presented in bold.
P4 *lmo1083*-sub	GGGG<u>TCTAGA</u>CGTAAATCATGACCAAGACGATCTTC	Reverse primer for the downstream region of *lmo1083*-sub. XbaI restriction site is underlined.
P5 *lmo1083*-sub	CGATGCGGGAATTGCCTAC	Forward primer for chromosomal validation of *lmo1083*-sub.
P6 *lmo1083*-sub	CCATTCGATAGTTTCTTTAATTCC	Reverse primer for chromosomal validation of *lmo1083*-sub.
P1 Δ*lmo1079*	GGGG<u>GAATTC</u>CAGGTACAGAGTCGAAATGG	Forward primer for the upstream flanking region of *lmo1079*. EcoRI restriction site is underlined.
P2 Δ*lmo1079*	AACATCTTCCATTAACTTTCTCCC	Reverse primer for the upstream flanking region of *lmo1079*.
P3 Δ*lmo1079*	**GGGAGAAAGTTAATGGAAGATGTT**CGCAAAAATAAAACACAATAAATGAGGG	Forward primer for the downstream flanking region of *lmo1079*. Anneals with P2 Δ*lmo1079* presented in bold.

P4 Δ*lmo1079*	CCCCGGATCCCGGTCATATTTGGACGTGTTTC	Reverse primer for the downstream flanking region of *lmo1079* sub. BamHI restriction site is underlined.
P5 Δ*lmo1079*	TGCGTCTCCAAGTCCCTTAG	Forward primer for validation of chromosomal Δ*lmo1079*.
P6 Δ*lmo1079*	CGCGTTCAAATGTTTCAGGG	Reverse primer for validation of chromosomal Δ*lmo1079*.
P1 Δ*lmo1083*	GGGGGAATTCCAAACGAACCCAAAAGCACAG	Forward primer for the upstream flanking region of *lmo1083*. EcoRI restriction site is underlined.
P2 Δ*lmo1083*	TACTAATAAATTCATTTATTTATTCTCCC	Reverse primer for the upstream flanking region of *lmo1083*.
P3 Δ*lmo1083*	**GGGAGAATAAATAAATGAATTTATTAGTA**GCAAAGTTGGGTGAATAAATATGAGT	Forward primer for the downstream flanking region of *lmo1083*. Anneals with P2 Δ*lmo1083* presented in bold.
P4 Δ*lmo1083*	CCCCGGATCCCTCCTGCTAATTTAGCAATTCC	Reverse primer for the downstream flanking region of *lmo1083* sub. BamHI restriction site is underlined.
P5 Δ*lmo1083*	CCAAAAGTGTTTGGAGATCATCG	Forward primer for validation of chromosomal Δ*lmo1083*.
P6 Δ*lmo1083*	CAGCTAGTTTAAGCATACTGTATAC	Reverse primer for validation of chromosomal Δ*lmo1083*.
	**NB probes**	
hly	CCATCTTTGTAACCTTTTCTTGG	Single stranded probe for *hly* mRNA
16S	GGCCATTACCCTACCAACTAGCTAATGCAC	Single stranded probe for *16S* mRNA
